# A qualitative study on acceptable levels of risk for pregnant women in clinical research

**DOI:** 10.1186/s12910-017-0194-9

**Published:** 2017-05-15

**Authors:** Indira S. E. van der Zande, Rieke van der Graaf, Martijn A. Oudijk, Johannes J. M. van Delden

**Affiliations:** 1 Julius Center for Health Sciences and Primary Care, Department of Medical Humanities, University Medical Center Utrecht, Utrecht, P.O. box 85500, 3508 GA The Netherlands; 2 Academic Medical Center, Department of Obstetrics and Gynaecology, Amsterdam, The Netherlands

**Keywords:** Risk, Pregnant women, Clinical research, Research ethics, Qualitative research

## Abstract

**Background:**

There is ambiguity with regard to what counts as an acceptable level of risk in clinical research in pregnant women and there is no input from stakeholders relative to such research risks. The aim of our paper was to explore what stakeholders who are actively involved in the conduct of clinical research in pregnant women deem an acceptable level of risk for pregnant women in clinical research. Accordingly, we used the APOSTEL VI study, a low-risk obstetrical randomised controlled trial, as a case-study.

**Methods:**

We conducted a prospective qualitative study using 35 in-depth semi-structured interviews and one focus group. We interviewed healthcare professionals, Research Ethics Committee members (RECs) and regulators who are actively involved in the conduct of clinical research in pregnant women, in addition to pregnant women recruited for the APOSTEL VI case-study in the Netherlands.

**Results:**

Three themes characterise the way stakeholders view risks in clinical research in pregnant women in general. Additionally, one theme characterises the way healthcare professionals and pregnant women view risks with respect to the case-study specifically. First, ideas on what constitutes an acceptable level of risk in general ranged from a preference for zero risk for the foetus up to minimal risk. Second, the desirability of clinical research in pregnant women in general was questioned altogether. Third, stakeholders proposed to establish an upper limit of risk in potentially beneficial clinical research in pregnant women in order to protect the foetus and the pregnant woman from harm. Fourth and finally, the case-study illustrates that healthcare professionals’ individual perception of risk may influence recruitment.

**Conclusions:**

Healthcare professionals, RECs, regulators and pregnant women are all risk adverse in practice, possibly explaining the continuing underrepresentation of pregnant women in clinical research. Determining the acceptable levels of risk on a universal level alone is insufficient, because the individual *perception* of risk also influences behaviour towards pregnant women in clinical research. Therefore, bioethicists and researchers might be interested in changing the perception of risk, which could be achieved by education and awareness about the actual benefits and harms of inclusion and exclusion of pregnant women.

## Background

Underrepresentation of pregnant women in clinical research has led to a lack of evidence-based knowledge on drugs and treatments, resulting in suboptimal care or even under-treatment of pregnant women and their foetuses [1–4]. In recent years, bioethicists, pharmacologists and regulators have therefore argued that research participation of pregnant women is essential in order to achieve fair healthcare opportunities for pregnant women and their future children [2, 5–10]. Various stakeholders have taken up the challenge of inclusion, for example by endorsing the view that pregnant women are presumed to be eligible for participation in clinical research [5, 9, 11]. Another (indirect) example can be found in the US Food and Drug Administration (FDA). Previously, it used the five pregnancy categories for drug-use in pregnant women, but after much critique that the categorisation was both over-simplistic and ambiguous [12, 13], it has now been replaced by the Pregnancy and Lactation Labelling Rule (PLLR), designed to improve risk versus benefit assessment of drugs used in pregnant and nursing mothers. Although the Final Rule is applauded by many for its effort to improve maternal care and help healthcare professionals to adequately treat pregnant women [12, 14, 15], it is also likely to further expose how little human data exist for most drugs that are available in the United States (92.9% of pharmaceutical drugs obtain pregnancy data from animal studies; 5.2% have human pregnancy data) [12, 16, 17]. Some have articulated the hope that the new labelling will provide added incentives for the development and conduction of more clinical research in pregnant women [14].

However, research participation of pregnant women is a complex matter and certain difficulties remain unresolved. One of these issues concerns the ambiguity with regard to what counts as an acceptable level of risk in clinical research in pregnant women. Currently, what may count as an acceptable level of risk can often not clearly be deduced from ethical guidelines or regulations, or the information that is provided is conflicting. The US Code of Federal Regulations (CFR) is one of the few places in which the risks to pregnant women are addressed. According to the Common Rule, the risk to the foetus should be “the least possible for achieving the objectives of the research” (minimising risk) and in research that has no potential individual benefit the risks should “not be greater than minimal” (45 CFR 46). Contrarily, the new CIOMS draft guideline proposes that when the social value of the research for pregnant or lactating women or their foetus or infant is compelling, a minor increase above minimal risk might be permitted in research that has no potential for individual benefit (CIOMS draft 2015). One could expect that the proposed broader phrasing of the CIOMS draft guideline might allow for more clinical research than was previously conceivable.

At the same time, the literature indicates that stakeholders such as Research Ethics Committees (RECs) or researchers or clinicians might be hesitant to conduct research in pregnant women [18, 19]. For example, it has been suggested that RECs often interpret guidance on research in pregnant women in an overly cautious manner and act as gatekeepers to research [5, 20, 21]. In the scarce literature on the willingness of pregnant women to participate in research it is seems that they themselves are willing to participate for different reasons, for example because of altruistic or personal motives [22–25]. However, these assumptions about pregnant women’s willingness are often based on hypothetical or retrospective research, while prospective research on their willingness is lacking. Moreover, there is no data on stakeholders views relative to research risks, while their input is essential in order to create clarity about acceptable levels of risk. Gaining an understanding of existing views in the field is not only of interest to the research community, it could also direct guideline committees and researchers in their development of general strategies on acceptable levels of risk in pregnant women. The aim of our paper was therefore to explore what stakeholders who are actively involved in the conduct of clinical research in pregnant women deem an acceptable level of risk for pregnant women in clinical research, by way of a qualitative approach.

## Methods

### Study design

We employed a qualitative study design using semi-structured interviews and one focus group to explore stakeholders’ views on the topic of acceptable risks for pregnant women in clinical research.

### Sample and setting

We sought to reach maximum variation in context and conducted the study among a variety of stakeholders whom were contacted by the researcher. We explored the topics through interviews with four groups: healthcare professionals, REC members, regulators and pregnant women. The healthcare professionals and REC members were recruited from two academic hospitals in the Netherlands, the University Medical Center Utrecht (UMC Utrecht) and the Academic Medical Center (AMC) in Amsterdam. We interviewed gynaecologists (*n* = 3), gynaecologists-in-training (*n* = 6), (research) midwifes (*n* = 5), and REC members (*n* = 5). Of the five REC members, two were also gynaecologists themselves. We also organised one focus group with regulators (*n* = 5) from LAREB, a Dutch pharmacovigilance centre where we spoke with employees from the Teratogenic Information Service (TIS) department. The focus group lasted 1:15h. In addition, we interviewed two regulators from the Dutch Medicine Evaluation Board (MEB).

Finally, we recruited pregnant women (*n* = 14) from the two previously mentioned academic hospitals in our qualitative study. Pregnant women were eligible when they were recruited for the APOSTEL VI study and had made their decision about enrolment in that study (see Table 1).

**Table 1:** Case-study: APOSTEL VI

The APOSTEL studies are a series of studies in the field of treatment of preterm labour within the Dutch Consortium for Healthcare Evaluation and Research in Obstetrics and Gynecology (NVOG Consortium 2.0.). The APOSTEL VI study in particular assesses whether a cervical pessary prolongs pregnancy in women who have been admitted for threatened preterm birth but remained undelivered after 48 h (http://www.studies-obsgyn.nl/apostel6). Women are randomly allocated to receive either a cervical pessary or no intervention. Women participating in the study were not perceived to be at an increased risk since previous studies using the pessary had shown no foetal adverse effects and the cervical pessary was not associated with increased neonatal or maternal morbidity and mortality (APOSTEL VI Research Protocol).
The APOSTEL VI study took place from November 2013 until September 2016, when the study was prematurely stopped following the advice of the Data and Safety Monitoring Board (DSMB). The premature cancellation was due to the fact that after interim analysis the intervention was unlikely to improve outcome, and maternal side effects were often present in the intervention arm.
Our qualitative study took place from March 2015 till September 2016 and we reached saturation before the APOSTEL VI itself was cancelled. In all our interviews it was therefore assumed that the APOSTEL VI would be completed.

**Fig. 1:**
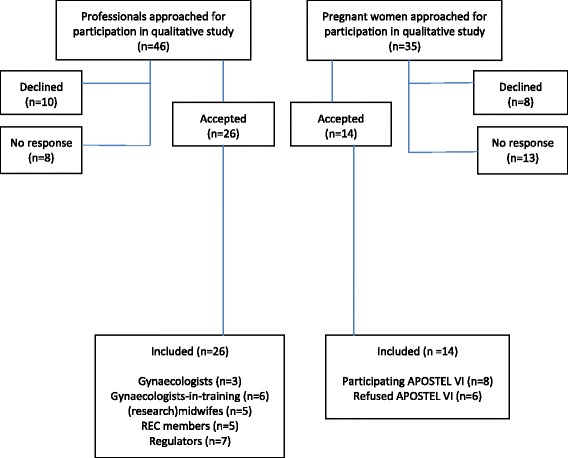
Flowchart of inclusions

We selected the APOSTEL VI study because it was the only obstetrical study in the Netherlands that at the time provided us access to the purposive sample of pregnant women recruited for a clinical study and the possibility to prospectively interview them. Accordingly, shortly after the women had decided about enrolment in the primary study, they were approached by research midwifes at the study sites. When they indicated an interest in our qualitative study they were later contacted by the researcher of the qualitative study and asked to participate in an interview. We interviewed the respondents after they were randomised to either perceive the pessary or no intervention. See Tables 2 and 3 with characteristics of participants and Fig. 1 with the flowchart of inclusion. The REC of the UMC Utrecht assessed the qualitative research proposal and issued a waiver for the project.

**Table 2:** Demographic characteristics professionals

Characteristics professionals	(*n* = 26) ^a^
Gender	
Male	11
Female	15
Age	
25 - 40	13
41 - 55	7
>55	6
Experience at present job (years)	
<5	13
5-10	6
11-15	4
16 - 20	3
Profession	
Gynaecologist	3
Gynaecologist-in-training^b^	6
Midwife^c^	5
REC member^d^	5
Regulator/knowledge centre	7

^a^5 regulators from the focus group, 21 interviewees

^b^1 gynaecologist-in-training was a gynaecologist-not-in-training (ANIOS)

^c^3 research midwifes from academic hospitals

^d^2 REC members were also gynaecologists

**Table 3:** Demographic characteristics pregnant women

Characteristics pregnant women	(*n* = 14)
Age	
<25	1
25 - 30	5
31 - 40	8
Parity	
Nulliparous	9
Primiparous	2
Multiparous	3
Gestational age (weeks)	
25 - 30	5
31 - 35	9
Education	
Highschool	3
Lower vocational (MBO)	3
College (HBO/WO)	4
Graduate degree	4
Partner	
Married	5
Living together	9
Single	0
Enrolment in study	
Participating in Apostel VI	8
*- Recruited from UMC Utrecht*	*3*
*- Recruited from AMC*	*5*
Not participating in Apostel VI	6
*- Recruited from UMC Utrecht*	*6*
*- Recruited from AMC*	*0*

### Data collection

All participants were interviewed by one researcher (IvdZ). The focus group was conducted by two researchers (IvdZ and RvdG). Verbal informed consent and written informed consent in case of the pregnant women was obtained from all participants. Initial interview topics and questions (Table 4) were formulated after examination of the relevant literature and discussion with members of the team. The semi-structured in-depth interviews were conducted according to a predefined topic list, however, according to the technique of constant comparative analysis, the interview topics evolved as the interviews progressed through an iterative process where the desired results is reached by repeating rounds of analysis [26]. In case of healthcare professionals and pregnant women. we used the APOSTEL VI study as a starting point to ask respondents about acceptable levels or risks, however, we then extended the conversation to cover questions about acceptable levels of risk in clinical research in pregnant women in general. Interviews took place at the workplace or the home of the respondents. Thematic saturation was reached after 20 interviews. Data collection took place from March 2015 to September 2016.

**Table 4:** General Topic List

• Balancing risks and potential benefits in general; • Whether there is a potential conflict between the mother and the foetus; • Whose interests should prevail; • Acceptable level of risks in certain types of research or in different phases; • Societal benefit versus therapeutic benefit; • Suggestions how to assess acceptability of risks; • Relation with acceptable research risks for children; • Balancing risks and potential benefits in the APOSTEL VI study; • Perceived risks of the APOSTEL VI study.	

### Data analysis

The analysis was carried out according to the thematic analysis method [27, 28]. The focus group and the interviews were transcribed verbatim and the data was imported in the software programme Nvivo 10 [29]. IvdZ independently coded the transcripts and through comparison across transcripts higher order themes were found. RvdG checked codes for consistency and the found themes were discussed at team meetings until a consensus was reached. To enhance the validity of our findings, we organised an expert meeting in the last phase of data collection. In the expert meeting we discussed our preliminary results and validated the data concerning the APOSTEL VI study.

## Results

Based on the responses of the respondents, we were able to identify three main themes characterising the stakeholders’ views on acceptable levels of risk in clinical research in pregnant women in general. Additionally, we identified one theme with respect to the APOSTEL VI case-study specifically. These themes emerged consistently within and across all interviews. Per theme, the views of the regulators, REC members, healthcare professionals and pregnant women are respectively presented. The first three themes concern observations based on the views of all respondents, while the theme relative to the APOSTEL VI study is based on the views of healthcare professionals and pregnant women specifically. Representative quotations were chosen in order to illustrate the identified themes (Tables 5 and 6).

**Table 5:** Representative quotations - Acceptable levels of risk in general

Theme	Quotations^a^
Continuum of acceptable risks in general	REG00, focus group LAREB: *But with regard to the foetus you want to accept nothing, risks have to be zero and you cannot guarantee that [..].* PW07, participating in APOSTEL VI: *There is never an acceptable risk for the foetus, never.* REC05, gynaecologist: *A pregnant woman is very much protected in our society. After all, a pregnant woman is a little sacred. I can understand that.* HCP09, gynaecologist: *You should at least demonstrate that you have no reason to assume that it [research] is unsafe.* HCP12, gynaecologist-in-training: *If you run the risk that if you stop with that medication the mother dies, that’s a different story than when you want an alternative for a very safe medication simply because the pills taste bad or they are big or whatever.* PW08, not participating in APOSTEL VI: *If you face a huge growth retardation and it will not change during the course of your pregnancy and you can participate in a study that potentially offers a remedy, then I think that I would also be more willing to go further […].* PW11, participating in APOSTEL VI: *The most important thing is whether there are risks for the baby. The baby needs to be able to grow optimally and survive the pregnancy. And as a mother I would accept quite a lot for that myself. Unless the risks are really dangerous [*e.g. *resulting in serious illness or death].*
Desirability of clinical research in pregnant women in general	REC01, legal expert: *When a researcher has already decided that he doesn’t want to expose a certain category of research subjects to the intervention or the medication or the risks of a study, well, then who am I as a REC member to tell him that maybe he should do that?* REC03, gynaecologist: *If it’s unnecessary than of course it’s always more sensible… Because that is something you notice, pregnancy always raises extra questions that make you think longer about whether it is acceptable or not. So for me I would say, let’s just keep them out if it is not strictly necessary to include them.* REG02, MEB member: *And it’s a question whether it always needs to be proven, because gathering the evidence requires a lot of pregnant women, with all the risks that entails.* HCP06, gynaecologist-in-training: *There is often so much happening when someone comes in and then you think, “oh yes, the trial. That is really the last priority.* HCP10, research midwife: *I said that I wouldn’t counsel for this study […]. You shouldn’t go beyond your own limits. I’m really not going to do something that I cannot support.* PW12, not participating in APOSTEL VI: *Why would you take a risk if you don’t have to, or if there is nothing to gain? I would not take such a risk for science.*
Interest in an upper limit of acceptable risk	HCP05, gynaecologist: *There should be a maximum risk for the foetus, but where do you draw the line?* HCP10, research midwife: *It worries me because if you as a caregiver offer this, and that woman is desperate enough and she thinks my child is going to die this is my last resort, then maybe she doesn’t look beyond delivering a child that is alive.* PW05, participating in APOSTEL VI: *They won’t allow you to take the big risks anyway. There are laws and regulations for that. […] It is offered for a reason and if they offer it, well than I guess that the risks won’t be so high.* PW11, participating in APOSTEl VI: *I trust that most studies are to some extend safe, they won’t allow you to take a lot of risk here [in the Netherlands]. That is a consideration that initially makes me say yes quite fast. Because if there is too much risk than it wouldn’t be conducted here.*

^a^Quotations are sometimes slightly modified in order to enhance readability

**Table 6:** Representative quotations - Acceptable levels of risk in APOSTEL VI specifically

Theme	Quotations^a^
Perceived risks of APOSTEL VI study	HCP06, gynaecologist-in-training: *A pessary is low risk, because you don’t have the connection with the child.* HCP03, gynaecologist-in-training: *We insert a device that is foreign to the body of which we know that it gives a local reaction, and if that is an inflammatory reaction it might just as well result in premature birth. So therefore it could also actually be a higher risk.* HCP04, gynaecologist-in-training: *Why would you do an intervention, why would we do something that has not been proven? I also wonder what the working mechanism of the pessary is, nobody can tell me, not even the big advocates.* HCP14, gynaecologist: *I don’t believe in the intervention* at all*, and luckily I don’t have to counsel for the study, but I do think that if you don’t know if something works the best way to find out is to conduct a study.* PW03, participating in APOSTEL VI: *They just don’t know if it [the pessary] results in an extended gestational time. But real risks, no I don’t think those were described.* PW14, participating in APOSTEL VI: *It’s very clear in the study that the risks are really very low, and that it won’t result in a premature birth which is the most important thing.*

^a^Quotations are sometimes slightly modified in order to enhance readability

**Table 7:** Overview of risk continuum

Stakeholder	What level of research risk? (for the foetus)	What type of research is acceptable?	When is research acceptable?
Regulators (LAREB)	Zero Zero Close to zero	None Observational Phase IV	Never Registries Post-authorisation studies with off-label medications already used by pregnant women
Regulators (MEB)	Zero Low but with exceptions	Observational Phase III and/or Phase IV	Registries Research that has potential individual benefit with high potential direct benefit for severely ill pregnant woman
RECs	Extremely low (below 1%)	Observational Intervention	Research that is not too demanding Research that has potential individual benefit with high potential direct benefit for severely ill pregnant woman
Pregnant women	Zero Minimal More than minimal	Observational Intervention	Not too demanding/useful for other women Research that has potential individual benefit with high potential direct benefit for the child
Healthcare professionals	Low, at least not higher in comparison to not participating	Observational Intervention Phase II Phase III	Research that has no potential individual benefit and research that has potential individual benefit Benefit for individual or group ”no harm in trying principle”

## Acceptable levels of risk in general

### Continuum of acceptable risks in general (Table 7)

The interviews demonstrated that regulators, REC members and pregnant women all start from the presumption of zero risk to the foetus. Nevertheless, the regulators from the pharmacovigilance centre were the ones most strongly adhering to the presumption. They said that clinical research that poses any risk should not be conducted and argued that when something is ‘research’, it automatically means that zero risk for the foetus cannot be unconditionally guaranteed and that risks should therefore be classified as high.

Interviews with regulators from the Medicine and Evaluation Board (MEB) and REC members demonstrated that they were willing to extend the level of acceptable research risks in case of research that has potential individual benefit, depending on the severity of the problem and the potential benefit. For example, REC members said that when zero risk is not attainable, the level of acceptable risk could be extended to “extremely low”, “below 1%” or “1:1.000.000”. Moreover, regulators from the MEB mentioned that inclusion of ill pregnant women in phase III trials with non-pregnant participants with a severe illness (such as rheumatic patients) would be acceptable because there would at least be knowledge about the effectiveness. Additionally, inclusion in phase IV post-authorisation studies (with medication originally labelled for different populations) was also suggested as an acceptable form of research in pregnant women with severe illnesses.

Pregnant women mentioned that they found the specific topic of weighing research risks very complex, but when further probed the initial answer “zero risk” changed in relation to different scenario’s concerning research that has potential individual benefit and research that has no potential individual benefit. In scenario’s where clinical research could potentially benefit the foetus, pregnant women mentioned that on behalf of the foetus they would consider participating in clinical research with higher risks (‘higher’ not further specified) than in clinical research with no potential personal benefit or than they would normally consider participating in.

During the interviews with healthcare professionals, it became clear that they, in their role as researchers, start from the presumption that pregnant women should be included in clinical research if there is a possibility for improvement of the current situation (for themselves or their group). Healthcare professionals specified the prerequisite for both observational and interventional research as follows: risks demonstrated to be to some extent foreseeable and low; a medication or intervention that is presumably safe and without long-term harmful effects; and a guarantee that women are not exposed to higher risks. The respondents mentioned that the prerequisites could be proven based on for example pre-clinical information, case-studies or database research. The basic assumption appeared to be that pregnant women in clinical research will either be better off, or that there is no effect. The tipping point of clinical research becoming unacceptable is when pregnant participants would have a chance of being *worse off.*

### Desirability of clinical research in pregnant women in general

The interviews with regulators and REC members showed that they generally understood the reasons why pregnant women are often excluded from all clinical research. These respondents actually questioned the need for inclusion. Concerns about potential risks as well as financial, ethical and methodological challenges were mentioned as underlying reasons. To illustrate, REC members explicated that although research that poses zero or negligible risks for the foetus would not be unacceptable, they still prefer not to conduct it because it is deemed unnecessary. Moreover, the interviews showed that REC members did not recognise a responsibility to ask researchers about exclusion of pregnant women, or they found such questions irrelevant. Some said they would advise the exclusion of pregnant women since, in their words, it is the easiest way to exclude such vulnerable groups. Instead, both regulators and REC members argued that investing in observational database research through registration systems is the preferred way to gather the necessary scientific knowledge.

The interviews with healthcare professionals demonstrated that they believed that clinical research in pregnant women is desirable in order to increase the evidence-base, although they did mention that researchers should in principle be more careful with pregnant women in comparison with non-pregnant research participants. When asked about inclusion of their own patients, healthcare professionals appeared to be more reluctant. Reasons that were mentioned were both practical (acute care has priority over clinical research) but also motivational, for example not believing a study is in the best interest of a patient or not believing in the intervention. Moreover, the healthcare professionals (as well as the regulators form the MEB) noticed that the lack of scientific knowledge concerning pregnant women is sometimes overrated or could be gathered in another way.

From the interviews with pregnant women, it became evident that their starting point in daily life was risk avoidance. For example, the women were careful with their food intake, they were extra cautious in traffic, and they would avoid taking any medication (including painkillers or natural vitamin supplements). The desire to avoid any risk for the foetus also extended to participation in clinical research, relative to which women reported that they would generally not participate in invasive clinical research because of potential risks. Research that would only pose risks to themselves and not to their foetus would be less problematic, similar to research that would encompass potential gain for themselves or the foetus. In relation to non-invasive research which posed no risks to the foetus (such as blood pricks or questionnaires), women reported an interest in participation in order to help other pregnant women.

### Interest in an upper limit of acceptable risk

Particularly in relation to acceptable risks in research that has potential individual benefit, the topic of an upper limit of risks emerged throughout the interviews. All respondents recognised the need for an upper limit of risks in light of possible harm to the foetus and potential misconceptions in research that has potential individual benefit, however, no one could explicitly stipulate a maximum. An example of an upper limit that was given was that in pregnant women one would never test a medication for safety. When respondents talked about potential misconceptions, they referred to their belief of pregnant women’s trust in the system and their idea that pregnant women would be willing to take excessive risks for their child. For instance, the healthcare professionals said that women appeared to have a somewhat excessive degree of intrinsic trust that their physician would never ask anything potentially harmful or not beneficial. And most women believed that research for which they as pregnant women were recruited or would be recruited for in the Netherlands would never actually expose them to any real risk in clinical research.

## Acceptable levels of risk in APOSTEL VI specifically

### Perceived risks of APOSTEL VI study

The interviews demonstrate that although the REC of the UMC Utrecht classified the APOSTEL VI study as a low-risk study, healthcare professionals’ opinions on the risk that the APOSTEL VI posed differed. Most healthcare professionals classified the APOSTEL VI as no risk (*n* = 4) or extremely low or low risk (*n* = 4), because the intervention is not a medication and the device is proven to be safe for the foetus and does not lead to increased risk during pregnancy. Other healthcare professionals classified the APOSTEL VI as a potential high risk study (*n* = 3), because there is not enough knowledge and the cervical pessary could actually affect the uterus in a negative way (e.g. by creating an inflammation which would then lead to preterm birth), thus comprising an increased risk. Others were unsure or had no opinion (*n* = 3). Furthermore, an overall scepticism with regard to the actual working mechanism of the pessary; concerns about the pessary itself (“it’s not nothing”/”it’s quite a thing”); and the extra internal exam (only for UMC Utrecht) surfaced throughout the conversations. But despite concerns about the study, the respondents mentioned that there was a distinction between “pointless” or “harmful” studies. Since the APOSTEL VI was not perceived to be harmful, in light of the current lack of knowledge on preventing preterm birth, most healthcare professionals were generally positive about inclusion of pregnant women in the APOSTEL VI study.

The interviews with pregnant women indicated that most perceived the APOSTEL VI to pose zero risk (*n* = 12) because enrolment would not negatively impact the development or growth of their child. The reasoning was that a pessary would not reach and therefore not affect the foetus (in contrast to e.g. a medication in the bloodstream), and that the device was safe because it was already used by other (pregnant) women. Moreover, the pregnant women mentioned that they found the burdens such as pain during the placement of the pessary and increased vaginal discharge relatively small. Two women who did not enrol in APOSTEL VI mentioned potential risk for the foetus as one of the reasons for not participating (because of the extra internal exam which they believed might cause a preterm birth), while one woman who did enrol also considered the risks to the foetus but ultimately decided to enrol because it would always be possible to remove the pessary.

## Discussion

Our qualitative study shows that among stakeholders who are actively involved in the conduct of clinical research in pregnant women in the Netherlands, risk-adversity is the main characteristic dominating the discourse on acceptable levels of risks. Risk-adversity is demonstrated in two ways. First, the risk-adverse attitude is so fundamentally present among stakeholders (including pregnant women themselves), that the need for the conduct of clinical research in pregnant women is questioned altogether. This possibly explains why pregnant women have even been excluded from research that posed no risk at all [2, 30, 31]. Correspondingly, stakeholders indicate a preference for zero risk for the foetus if research does take place. And, when zero risk is not achievable, stakeholders propose to establish an upper limit (not further specified) in potentially beneficial research in order to protect the foetus from harm and the pregnant woman from potential misconceptions about research participation. Currently, upper limits of risk are primarily set in particular types of research that has no potential individual benefit, with persons who are incapable of giving informed consent. However, for research with children and incompetents, no upper limits of risk are set when the research has the potential for individual benefit [32]. The interest in an upper limit for research that has the potential to benefit pregnant women is thus even more stringent for pregnant women than for research with persons who are incapable of giving informed consent. Since there is no immediately obvious reason why pregnant women would be incompetent to make a decision about research participation [van der Zande ISE, van der Graaf R, Oudijk MA, van Delden JJM. Vulnerability of Pregnant Women in Clinical Research. Journal of Medical Ethics. Forthcoming. 2017], the interest in an upper limit might be another illustration of stakeholder’s risk adversity towards clinical research in pregnant women.

Second, the risk-adverse attitude also influences the actual conduct of clinical research in pregnant women. At first, there appeared to be a difference between regulators, pregnant women and REC members on the one hand, and healthcare professionals on the other, where the latter seem more willing to include pregnant women for potential group benefits for their population. However, while healthcare professionals in their role as researcher report a willingness to advance inclusion of pregnant women in clinical research, in practice they are also reluctant to include their patients and sometimes even resort to gatekeeping, the fashion where eligible participants are prevented from entering research [4, 33, 34]. It appears that healthcare professionals make their individual judgements about risks and that they sometimes perceive minimal risk as high risk. The personal opinion of a study combined with the perception of risk seems to influence behaviour, as illustrated by our case-study. The now prematurely cancelled APOSTEL VI was originally classified as a low risk study, but it was actually rejected by a number of academic centres due to the perceived high risks that the intervention would pose. Moreover, healthcare professionals from centres where the case-study was approved made individual judgements on the risk and voiced various concerns with regard to the study, in our case doubts about the pessary as an intervention. A lack of equipoise concerning an intervention has been suggested earlier as a reason for hampering recruitment [35] (also suggested in relation to the APOSTEL IV study [36]). Moreover, it could also explain why even minimal risk studies often get cancelled. Cancellation can happen because of various reasons such as financial or safety issues, but also because of disappointing patient recruitment rates which might be traced back to gatekeeping by healthcare professionals [35] (APOSTEL IV study [36] and possibly also the APOSTEL VI study).

Bioethicists believe that more regulatory clarity on accepted levels of risk in clinical research in pregnant women may result in fair inclusion of pregnant women [5, 37]. While a universally accepted risk standard might indeed contribute to fair inclusion by taking away ambiguity with regard to what kind of research would be acceptable, our analysis shows that a classification of risk alone is not sufficient since the *perception* of risk also strongly influences behaviour. In order for universal risk standards to be applied in practice, bioethicists might therefore be interested in stimulating an alteration in the framework of thought on risk for pregnant women. A possibility would be to address the feasibility of a study beforehand, by aligning the risk classification between RECs and healthcare professionals. Additionally, educating REC members and healthcare professionals to internalise the content of present guidelines (most guidelines already allow for certain risks) and to equally focus on research benefits, next to risks, and on the need for evidence-based clinical care and treatment could be worthwhile [5, 19, 38]. Moreover, raising awareness about the actual need for clinical research in pregnant women could stimulate patient advocacy, which, as demonstrated by the increased conduct of research in children or certain orphan diseases after active involvement of patients, could be an effective method [39], also taking into consideration that pregnant women reported altruistic motives to participate in non-invasive studies with no risk to the foetus. Finally, guideline committees and researchers may want to take notice of the discrepancies about risk acceptability and the reigning precautionary principle when they develop further guidance on clinical research in pregnant women.

### Limitations

This qualitative study has a number of limitations. First, we interviewed stakeholders regarding only the Dutch situation and from an academic setting, it is possible that the results are different in other countries and other settings, thus challenging the generalizability of the findings. Second, we did not include any pharmaceutical companies in our stakeholder list. Since we realise that pharmaceutical companies are an important stakeholder we contacted seven organisations with a request to participate, but unfortunately we were unable to conduct any interviews since they did not respond or did not want to participate in our study. Third, the saturation number of twenty interviews was reached on a group level, but not always on sub-group level. For example, regarding the case-study we only interviewed healthcare professionals and pregnant women. As such, our inter-group comparisons are less valid than our group analyses. Finally, we only included pregnant participants who were enrolled in the APOSTEL VI study, a group that consists of women that become sick during their pregnancy. We selected the APOSTEL VI study because it was the only obstetrical study in the Netherlands that at the time provided us access to the purposive sample of pregnant women recruited for a clinical study and the possibility to prospectively interview them. Future research should also aim to include research subjects from the group of sick women who become pregnant and participants recruited for non-obstetrical studies. We attempted to include women from the latter group, but all three trials we collaborated with were unfortunately cancelled.

## Conclusions

Stakeholders generally deem clinical research in pregnant women only acceptable when the risks to the foetus are zero or very close to zero. Although there seems to be a conflict between healthcare professionals in their role as researchers (wanting to advance the interest of the group) and RECs, regulators and pregnant women (wanting to safeguard the interest of the individual), in practice everybody acts risk-adverse in the context of research. The risk-adverse attitude probably explains the continuing underrepresentation of pregnant women in clinical research. Consequentially, fair inclusion of pregnant women may not be achieved by determining the acceptable levels of risk alone, because the *perception* of risk also influences stakeholders’ behaviour. Therefore, bioethicists and researchers might be interested in changing the perception of risk, for example by education of professionals and by stimulating patient advocacy amongst pregnant women. In addition, guideline committees and researchers may want to take notice of the discrepancies about risk acceptability when they develop further guidance on clinical research in pregnant women.

## Abbreviations

AMC: Academic Medical Center Amsterdam
APOSTEL VI trial: Assessment of Perinatal Outcome after Specific Treatment in Early Labor
APOSTEL IV trial: Assessment of Perinatal Outcome by use of Tocolysis in Early Labor
CFR: Code of Federal Regulations
CIOMS: Council for International Organizations of Medical Sciences
DSMB: Data and Safety Monitoring Board
FDA: Food and Drug Administration
HCP: Healthcare professional
LAREB: Netherlands Pharmacovigilance Centre
MEB: Medicines Evaluation Board
NVOG: Dutch Consortium for Healthcare Evaluation and Research in Obstetrics and Gynecology
PLLR: Pregnancy and Lactation Labeling Rule (Final Rule)
PW: Pregnant woman
REC: Research Ethics Committee (member)
REG: Regulator
TIS: Teratology Information Service
UMC Utrecht: University Medical Center Utrecht
